# Mitochondrial Targeted Coenzyme Q, Superoxide, and Fuel Selectivity in Endothelial Cells

**DOI:** 10.1371/journal.pone.0004250

**Published:** 2009-01-22

**Authors:** Brian D. Fink, Yunxia O'Malley, Brian L. Dake, Nicolette C. Ross, Thomas E. Prisinzano, William I. Sivitz

**Affiliations:** 1 Division of Endocrinology and Metabolism, Department of Internal Medicine, Iowa City Veterans Affairs Medical Center and the University of Iowa, Iowa City, Iowa, United States of America; 2 Department of Medicinal Chemistry, University of Kansas, Lawrence, Kansas, United States of America; Karolinska Institutet, Sweden

## Abstract

**Background:**

Previously, we reported that the “antioxidant” compound “mitoQ” (mitochondrial-targeted ubiquinol/ubiquinone) actually increased superoxide production by bovine aortic endothelial (BAE) cell mitochondria incubated with complex I but not complex II substrates.

**Methods and Results:**

To further define the site of action of the targeted coenzyme Q compound, we extended these studies to include different substrate and inhibitor conditions. In addition, we assessed the effects of mitoquinone on mitochondrial respiration, measured respiration and mitochondrial membrane potential in intact cells, and tested the intriguing hypothesis that mitoquinone might impart fuel selectivity in intact BAE cells. In mitochondria respiring on differing concentrations of complex I substrates, mitoquinone and rotenone had interactive effects on ROS consistent with redox cycling at multiple sites within complex I. Mitoquinone increased respiration in isolated mitochondria respiring on complex I but not complex II substrates. Mitoquinone also increased oxygen consumption by intact BAE cells. Moreover, when added to intact cells at 50 to 1000 nM, mitoquinone increased glucose oxidation and reduced fat oxidation, at doses that did not alter membrane potential or induce cell toxicity. Although high dose mitoquinone reduced mitochondrial membrane potential, the positively charged mitochondrial-targeted cation, decyltriphenylphosphonium (mitoquinone without the coenzyme Q moiety), decreased membrane potential more than mitoquinone, but did not alter fuel selectivity. Therefore, non-specific effects of the positive charge were not responsible and the quinone moiety is required for altered nutrient selectivity.

**Conclusions:**

In summary, the interactive effects of mitoquinone and rotenone are consistent with redox cycling at more than one site within complex I. In addition, mitoquinone has substrate dependent effects on mitochondrial respiration, increases repiration by intact cells, and alters fuel selectivity favoring glucose over fatty acid oxidation at the intact cell level.

## Introduction

Mitochondria are likely the predominant source of reactive oxygen species (ROS) in many cell types [1]–[4]. This is underscored by recent evidence that mitochondrial oxidative damage may underlie problems including cell damage in degenerative diseases and aging [5]–[7], vascular disease, and complications of diabetes [8]–[16]. Given the vascular nature of these problems understanding the role of mitochondrial oxidative stress in endothelial cells is particularly important.

Concern over the contribution of ROS to vascular and degenerative disease, has led to attempts at antioxidant therapy. Efforts are now underway to develop effective antioxidant compounds targeted to mitochondria [17], [18]. In particular, targeted forms of coenzyme Q have attracted attention and are under development as therapeutic agents [19]. However, the effects of targeted CoQ on ROS production by mitochondria are still not well understood. Moreover, little is known of the metabolic consequences of loading mitochondria with Coenzyme Q (CoQ10) analogs.

In past studies of bovine aortic endothelial (BAE) cell mitochondria, we demonstrated that a mitochondrial targeted CoQ compound termed “mitoQ” (mitoquinone, mitoquinol, or a mixture of these two redox cycling compounds) markedly increased or decreased reactive oxygen species (ROS) generation depending on substrate provided for fuel [20]. MitoQ markedly increased superoxide production during forward electron transport in mitochondria respiring on the complex I (NADH∶ubiquinone oxidoreductase) substrates, glutamate plus malate. On the other hand mitoQ inhibited ROS generated by BAE mitochondria respiring on the complex II (succinate∶ubiquinone oxidoreductase) substrate, succinate, a condition wherein ROS production occurs through reverse electron transport or backflow of electrons to complex I originating from complex II.

During respiration on complex I substrates, superoxide appears to result from redox cycling of endogenous CoQ10 or exogenous analogs at Q-binding sites within complex I [20]–[22]. Recently a two site model for mitoquinone redox cycling in complex I was proposed with one site proximal and one distal to the site of rotenone inhibition at the N2 iron-sulfur cluster [21]. Our previous work showed that the effect of mitoquinone on ROS production was rotenone sensitive [20]. Therefore, in this work we examined ROS production as affected by different relative concentrations of mitoquinone and rotenone to determine whether simple dose dependent effects or more complex interactions were involved, which might be better explained by a two-site model.

Further, and perhaps of greater importance, we addressed the issues of whether mitoquinone might induce a substrate specific increase in mitochondrial oxygen consumption (as might be expected given the substrate specific effects on ROS) and whether this might be associated with alterations in cellular respiration and nutrient selectivity. This could follow based on the following reasoning. Complex I substrates generate NADH which donates electrons to complex I. In contrast, electron donation at other entry sites including complex II and the ETF-ubiquinone reductase requires FADH_2_. Glucose oxidation through glycolysis and pyruvate dehydrogenase to acetyl CoA generates NADH. In contrast, fatty acid oxidation to acetyl-CoA generates FADH_2_ through the process of β-oxidation. Acetyl CoA from both sources feeds the TCA cycle. So, although mitochondrial fatty acid β-oxidation is more efficient in terms of total reducing equivalents generated per molecule, glucose oxidation compared to fat oxidation generates proportionally more NADH (as a percent of total reducing equivalents generated). So, we reasoned that if mitoquinone were more effective at generating oxygen radicals on substrates that produce NADH (complex I substrates) than those which generate FADH_2_, then mitoquinone might also increase respiration on complex I substrates more than complex II substrates. If so, mitoquinone might favor metabolism of nutrients that generate proportionately more NADH than FADH_2_ which might then translate to proportionately more (in the presence of mitoquinone versus absence) use of glucose than fat.

The issue of fuel selectivity is of high interest in the fields of diabetes, obesity, and associated cardiovascular disease. In fact lack of metabolic “flexibility” such as the capacity to switch between fat and glucose oxidation has been implicated in cardiac complications of diabetes [23], [24] and in the pathogenesis of insulin resistance as seen in type 2 diabetes and obesity [25].

Here we report novel information covering two important issues. First we provide new data on the interactions of mitoquinone and rotenone at different concentrations of complex I substrates and discuss the implications for redox cycling at complex I. Second, we provide novel data demonstrating that mitoquinone does in fact alter respiration and fuel selectivity by intact cells. Finally we consider the effects of mitoquinone on mitochondrial membrane potential and whether such effects might affect fuel selectivity and/or cell toxicity.

## Materials and Methods

### Reagents and supplies

Mitoquinone and mitoquinol were synthesized from commercially available 11-bromoundecanoic acid and 2,3-dimethoxy-5-methyl-1,4-benzoquinone as described [26], [27]. Other reagents, kits, and supplies were as specified or purchased from standard sources.

MitoQ consists of the quinone/quinol moiety of endogenous coenzyme Q, linked to a 10 carbon side chain, covalently bound to triphenylphosphonium. Control compounds included endogenous coenzyme Q, decylTPP consisting of triphenylphosphonium and the 10 carbon chain of mitoQ but without the quinone moiety, and decylQ consisting of the carbon chain and quinone/quinol moiety but without the targeting cation. The molecular structures of these compounds are included in supplementary material (Methods S1).

Primary antibodies included mouse monoclonal anti-porin (A21317, Invitrogen, Carlsbad, CA), goat polyclonals anti-actin and anti-histone deacetylase-1 (HDAC1)(sc-1615 and sc-6298, respectively, Santa Cruz Biotechnologies, Santa Cruz, CA), and rabbit anti-catalase IgG (01-05-030000, Athens Research and Technology, Athens, GA). Secondary antibodies consisted of goat anti-mouse, donkey anti-goat, and goat anti-rabbit (Santa Cruz).

#### Cell culture

BAE cells were grown in medium M199 (Invitrogen) supplemented with minimal essential medium amino acids (Invitrogen), penicillin/streptomycin (Invitrogen), minimal essential medium vitamins (Sigma), and 20% fetal bovine serum (HyClone, Logan, UT) as described [28]. Cells were grown to near confluence in 150-cm^2^ flasks and used between passages 6 and 12.

### Isolation of Mitochondria

Cells were washed with phosphate buffered saline (PBS) and scraped. Collected cells were homogenized using a Dounce homogenizer in ice-cold homogenization buffer (0.25 M sucrose, 5 mM HEPES, 0.1 mM EDTA, pH 7.2) with 0.1% fatty acid-free bovine serum albumin (BSA). The homogenate was centrifuged at 1000×g for 10 min. The pellet was discarded and the supernatant was centrifuged again at 10,000×g for 10 min to obtain the mitochondrial pellet. The resulting pellet was then washed three times in homogenization buffer without BSA and resuspended in media as described below. Protein was determined by the Bradford method (BioRad, Hercules, CA). Mitochondria prepared in this way were of good quality as documented by an increase in respiratory rate of 3.5–6 fold after addition of carbonyl cyanide p-[trifluoromethoxy]-phenyl-hydrazone (FCCP) as an uncoupler.

Purity of mitochondria prepared in this way was documented by assessing mitochondrial and intact cell expression of the mitochondrial protein, porin; the cytoplasmic protein, catalase; the nuclear protein, HDAC1; and actin by immunoblot analysis. In our mitochondrial studies, all individual experiments (each n of 1) compare conditions within the same preparation, so the extent of purity would be the same for all conditions.

### Immunoblotting

Ten µg protein per lane were loaded and separated by 12.5% polyacrylamide gel electrophoresis and electroblotted to nitrocellulose membranes (Millipore Corp., Bedford, MA). For porin, blots were incubated with mouse anti-porin, 1∶25,000, in TTBS (tris buffered saline, pH 7.6 with 1 ml/L TWEEN 20)/2.5% BSA overnight at 4°C. For actin, blots were blocked with TTBS/5% milk at RT for 1 h and incubated in goat anti-actin, 1∶2000, in TTBS/2.5% BSA overnight at 4°C. For HDAC1, blots were incubated in goat anti-HDAC1, 1∶500, in TTBS/2.5% BSA overnight at 4°C. For catalase, blots were incubated in rabbit anti-catalase, 1∶2500 in TTBS/2.5% BSA overnight at 4°C. Following primary antibody incubations, blots were washed in TTBS, and incubated in secondary Ab, 1∶20,000, for 1 hour at room temperature in TTBS/5% milk. After secondary antibody incubations, blots were washed with TTBS and developed by enhanced chemiluminescence using a standard kit (ECL, Amersham Pharmacia Biotech, Piscataway, NJ).

### Mitochondrial respiration

Mitochondrial respiration was measured as we previously described [20], [29] using a Clark miniature oxygen electrode and small (0.6 ml) volume chamber with stir bar (Instech Laboratories, Inc., Plymouth Meeting, PA) at 37°C in ionic respiratory buffer (120 mM KCl, 5 mM KH_2_PO_4_, 2 mM MgCl_2_, 1 mM EGTA, 3 mM HEPES, pH 7.2 with 0.3% fatty acid free BSA). Isolated mitochondria (0.5 mg protein/ml) were incubated in the respiratory media and oxygen consumption quantified. To determine state 4 and state 3 respiration, oxygen consumption was continuously recorded with sequential additions of 5 mM succinate, 0.2 mM ADP, and finally 2.5 µM carbonyl cyanide p-[trifluoromethoxy]-phenyl-hydrazone (FCCP) to induce maximal chemical uncoupling using standard methodology as we have employed in the past [30]. The ADP∶O ratio was determined in standard fashion as we have carried out in the past [30], calculated as ADP added divided by oxygen consumed during the duration of state 3 respiration

### Mitochondrial ROS production

Mitochondria were studied during state 4 respiration, under which circumstance oxygen radical formation is enhanced as electron flow leads to high potential unmitigated by ATP generation [31]. H_2_O_2_ production was assessed as we previously described [20] using the fluorescent probe 10-acetyl-3,7-dihydroxyphenoxazine (DHPA) (Amplex Red, Invitrogen, Carlsbad, CA). In the presence of horseradish peroxidase, DHPA reacts with H_2_O_2_ to generate the fluorescent compound resorufin. ROS detected in this way derives largely from superoxide converted to H_2_O_2_ by matrix superoxide dismutase and released externally. As we previously showed by inhibitor analysis and by comparison to complex III superoxide detected by electron paramagnetic resonance spectroscopy (EPR) [20], and as shown by others [32], H_2_O_2_ detected in this way largely derives from superoxide released by complex I.

Samples were prepared in 96-well plates containing 0.06 ml per well of respiratory buffer. Fluorescence was measured as we previously described [20] once every 60 seconds and carried out for 30 cycles. For quantification, a H_2_O_2_ standard curve ranging from 0–12 µM was prepared and included on each plate. Addition of substrates, mitoquinone, or diphenyleneiodinium chloride (DPI) to respiratory buffer did not affect the H_2_O_2_ standard curve. Addition of rotenone at 5 µM (maximum dose used) slightly raised the height of the standard curve at all points with no change in slope so the data were corrected for this factor. The lower doses of rotenone utilized in this work had no effect on the standard curve.

Addition of catalase, 500 units/ml, reduced fluorescence to below the detectable limit indicating specificity for H_2_O_2_. Addition of the ETS inhibitors rotenone or DPI or tested quinone or control compounds to mitochondria in the absence of substrate altered fluorescence no more than 5%.

### Respiration by intact BAE cells

We used a method adapted from perfusion methodology designed to quantify oxygen use by cells under microscopy [33]. Here we grew BAE cells on glass microcarrier beads (Cytodex, Sigma Life Sciences, Inc.) enabling perfusion on a column in bead volumes of 1.0–1.5 ml at flow rates of 1–1.5 ml/min. Respiration was quantified as the steady state difference in oxygen tension determined between electrodes placed proximal and distal to the column. The electrodes could be precisely calibrated to each other by shunting medium around the perfusion column through a control bead column without cells. Oxygen use per volume of beads perfused is given by the difference in oxygen tension and known flow rate[(% drop in O_2_ content across the column×oxygen content at atmospheric pressure (nmol/ml)×flow rate (ml/min)]/bead volume (ml). The apparatus and a sample run are depicted in supplementary material (Methods S1).

Oxygen consumption depends on bead volume, flow rate, and the number of cells per bead volume. Bead volume and flow rates were identical for each of three experiments comparing mitoquinone to control (decylQ). Although we did not quantify the number of cells per bead volume the same preparations were used for mitoquinone and control in each experiment so any variation with cell number would be identical for mitoquinone and control. An example run is shown in supplemental material (Methods S1) wherein FCCP was added at the end demonstrating the expected transient increase in O2 consumption with chemical uncoupling.

### Glucose and Oleate oxidation

Cells were washed and then preincubated for 20 min in culture medium with 5.5 mM glucose, and either 10 µM or 200 µM oleate with 1.5% fatty acid-free BSA, and 1 mM carnitine in 12-well plates (Costar, Corning Inc., Acton, MA) containing 0.6 ml total volume per well. MitoQ (1 µM) or control compounds at the same concentration were added at the beginning of the 20 min preincubation (time −20 min) before addition of label at time 0. Separate groups of experiments were performed in the presence of oleate, 200 µM, and oleate, 10 µM. In each group of experiments glucose and oleate oxidation were assessed in parallel studies under the same conditions except for the addition of either [1–14C]oleic acid or D-[14C(U)]glucose. Cells were incubated for 120 min before trapping of CO_2_ released by perchloric acid as we previously described [30]. Final specific activities in the incubation media were 1.52×10^6^ µCi/pmol for cold oleate at 200 µM (total oleate with label 206 µM) and 20.1 µCi/pmol for cold oleate at 10 µM (total oleate with label 15.7 µM). Final specific activity of glucose was 0.0463×10^3^ µCi/nmol for cold glucose at 5.5 mM (total glucose with label 5.6 mM).

An additional group of experiments were carried out to determine the dose-response characteristics of the mitoquinone on fuel selectivity. These experiments were preformed in the same fashion except that cells were exposed to various concentrations of mitoquinone or vehicle in culture medium overnight (18 h) before addition of [1–14C]oleic acid or D-[14C(U)]glucose and 50 µM cold oleate at time zero (before the 120 minute incubation with label). Final specific activity of oleate was 5.61×10^6^ µCi/pmol (total oleate with label 56 µM). Final specific activity of glucose was 0.0463×10^3^ µCi/nmol (total glucose with label 5.6 mM).

### Mitochondrial membrane potential

Cells were exposed to various doses of mitoquinone, decylTPP, CoQ10, or vehicle in culture medium overnight for 18 h. The potential sensitive probe, JC-1 was then added at a concentration of 0.5 µg/ml for one hour and washed with Earle's balanced salt solution before determining red and green fluorescence at excitation and emission wavelengths 544/590 and 485/520 respectively. The red to green ratio was calculated as an index of mitochondrial potential.

### Cell toxicity assays

Toxicity was assayed by two methods. First, the ability of cells to convert the redox dye resazurin into resorufin, a property lost by cells as metabolic activity is impaired [34], was determined using a kit (CellTiter-Blue, Promega). BAE cells were treated overnight with mitoquinone or control compounds prior to end point addition of the resazurin reagent for three h. Conversion of resorufin was determined by fluorescence at excitation of 560 nm and emission at 590. Second we assayed cell membrane integrity by measuring LDH released into the culture medium in comparison to a standard curve. To prepare the curve, BAE cells were fully lysed and pooled to generate an LDH concentration reflective of 100% lysis and serial dilutions made generating a curve representing LDH (fluorescence) versus estimated percent lysis. Background fluorescence (about 9% of total) was subtracted in each assay. The assay was carried out using a kit (CytoTox-ONE, Promega) according to the manufacturer's recommended protocol.

### Statistics

Data were analyzed by ANOVA with post-tests as indicated.

## Results

### Purity of BAE cell mitochondria

**Figure 1:**
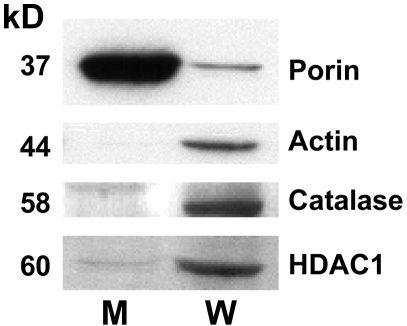
Mitochondria were highly purified as demonstrated by the relative expression of the mitochondrial specific protein, porin; the cytoplasmic protein, catalase; the nuclear protein, HDAC1; and actin in mitochondrial (M) and whole cell (W) fractions. Extracts were subject to immunoblot analysis using the antibody to the indicated protein. Findings are representative of four different mitochondrial preparations.

BAE mitochondria were highly pure as shown by the relative expression of the mitochondrial specific protein, porin; the cytoplasmic protein, catalase; the nuclear protein, HDAC1; and actin in mitochondrial and whole cell fractions (figure 1). It is possible that there is very minor contamination of mitochondria with nuclear particles based on a faint HDAC1 signal in the mitochondrial fraction.

### Substrate dependent effects of mitoQ on mitochondrial ROS production

**Figure 2:**
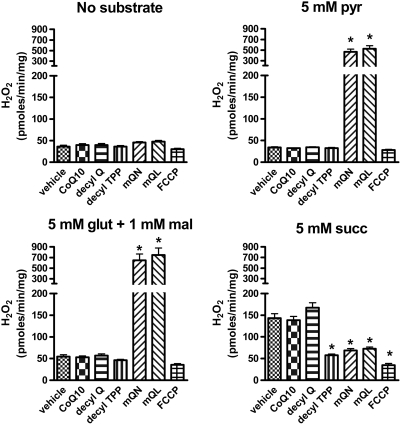
Effect of mitoquinone (mQN) and mitoquinol (mQL) compared to control compounds on H_2_O_2_ production by isolated BAE mitochondria fueled by different substrates. Mitochondria were incubated in respiratory buffer under state 4 conditions fueled by pyruvate (pyr), glutamate (glut)+malate (mal), or succinate (succ) as indicated above the individual graphs. Added compounds (1.0 µM), vehicle, or FCCP (2.5 µM) were present as indicated on the X-axis. Data represent mean±SE, * p<0.01 compared to vehicle by ANOVA. n = 6 mitochondrial preparations for all data points. Each individual value represents the mean of two replicate wells.

Consistent with our previous findings [20], mitoQ markedly increased H_2_O_2_ production in the presence of the complex I substrates, glutamate and malate, while reducing H_2_O_2_ production in the presence of the complex II fuel, succinate. Here we show similar findings (figure 2) when mitochondria were incubated in the presence of pyruvate which can also donate electrons to complex I through NADH. Tested compounds demonstrated minimal non-specific fluorescence as evidenced by the data generated in the absence of substrate.

### Substrate dependent effects of mitoQ on mitochondrial respiration

**Figure 3 pone-0004250-g003:**
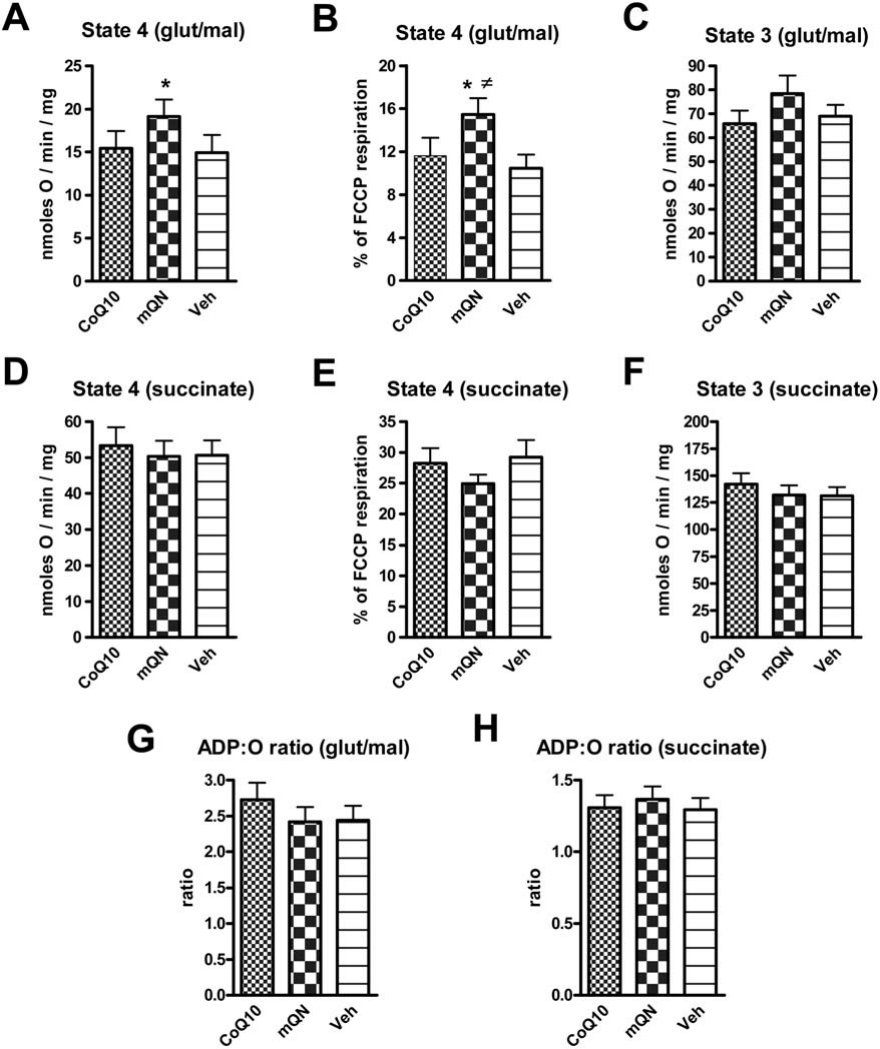
Effect of mitoquinone (mQN) compared to CoQ10 and vehicle on respiration by isolated BAE mitochondria fueled by different substrates. Mitochondria were incubated under state 4 and state 3 conditions fueled by 5 mM glutamate plus 1 mM malate (panels A, B, C) or 5 mM succinate (panels D, E, F). Mitochondria were exposed to 1.0 µM CoQ10, 1.0 µM mitoquinone (mQN), or vehicle (Veh) as indicated. Respiration is expressed in absolute terms (panels A,C,D,E) or as percent of maximal respiration induced by 2.5 µM FCCP (panels B and E). The ADP∶O ratio is depicted in panels G and H. Data represent mean±SE, * p<0.05 compared to CoQ10 or vehicle (panel A) or to CoQ10 (panel B), ≠ p<0.01 compared to vehicle (panel B) by ANOVA, n = 13 for all incubations.

Figure 3 shows the effect of mitoquinone, compared to CoQ10 or vehicle, on respiration in isolated BAE mitochondria determined by polarography using a Clarke electrode to measure O2 utilization. These data show that mitoquinone significantly increased state 4 respiration by isolated BAE mitochondria respiring on the complex I substrates, glutamate and malate. Mitoquinone resulted in a non-significant decrease in respiration on the complex II fuel, succinate. Essentially, the same results were observed when respiration was expressed as a percentage of maximal respiration determined in the presence of the chemical uncoupler FCCP. Mitoquinone resulted in similar, but non-significant, alterations in state 3 respiration. Mitoquinone did not significantly alter the ADP∶O ratio in mitochondria respiring on complex I or Complex II substrates.

### ROS production in the presence of complex I inhibitors

**Figure 4:**
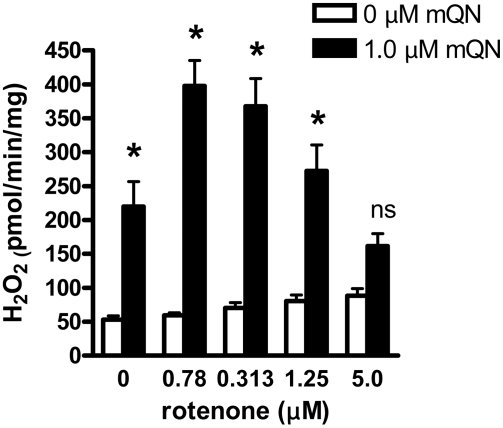
Interactive effect of rotenone and mitoquinone on H_2_O_2_ production by BAE mitochondria fueled by 5 mM glutamate plus 1 mM malate incubated in respiratory buffer under state 4 conditions. Mitochondria were exposed to mitoquinone (mQN) and/or rotenone at the concentrations indicated. Data represent mean±SE, n = 4 for each data point. * p<0.001 or non-significant (ns) compared to 0 µM mQN by 2-way ANOVA (factors are mQN and rotenone). There was significant interaction (p<0.001) between factors indicating the effect of mQN is dependent on the rotenone concentration.

In pilot experiments, we noted that rotenone inhibits the effect of mitoquinone on ROS in the presence of complex I substrates. However the effect seemed to change as different doses of these compounds were tested. Figure 4 depicts the effect of mitoquinone at different concentrations of rotenone on H_2_O_2_ production by BAE mitochondria fueled with complex I substrates. As shown, the effect of increasing the rotenone dose is biphasic, first increasing then decreasing mitoquinone induced ROS, thus generating the inverse U-shaped relationship. By two-way ANOVA, the data are highly interactive (p<0.001) indicating that the effect of mitoquinone is highly dependent on the rotenone concentration.

**Figure 5:**
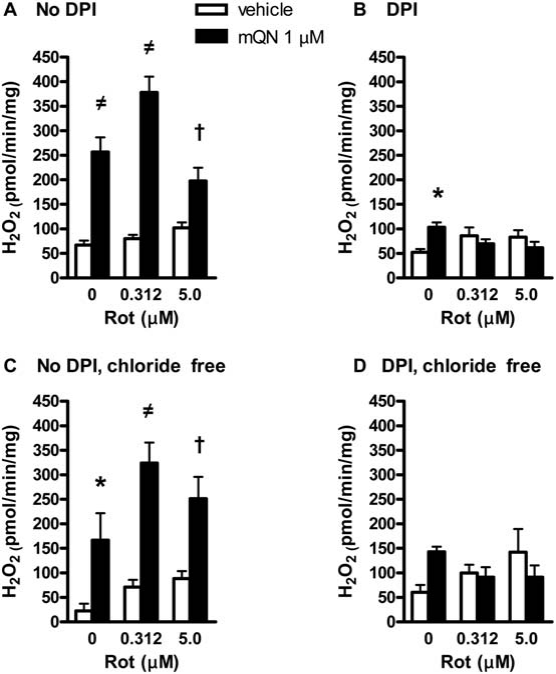
Effect of diphenyleneiodinium chloride (DPI) on H_2_O_2_ production by BAE mitochondria as affected by mitoquinone (mQN) at different doses of rotenone (Rot). Mitochondria were fueled by 5 mM glutamate plus 1 mM malate and incubated as in figure 4. Panel A) Mitochondria were incubated in respiratory buffer with no additions. Panel B) Addition of 5 µM DPI. Panel C) Mitochondria were incubated in chloride free respiratory buffer (gluconate substituted for chloride) with no additions. Panel D) Addition of 5 µM DPI in chloride free buffer. Data represent mean±SE, n = 10 in panels A and B, n = 6 in panels C and D. * p<0.05, † p<0.01, ≠ p<0.001 for mitoquinone compared to absence of the compound. Data were analyzed by 2-way ANOVA (factors mQN or Rot). Interaction was significant in panels A, B, and D.

In other experiments, we examined the effect of 5 µM DPI, which inhibits the FMN site of complex I, on ROS production as affected by mitoquinone at different rotenone concentrations. As shown, DPI reduced the effect of mitoquinone at all doses of rotenone. Since, there is evidence that DPI may also act on mitochondrial chloride channels [35], we tested the effect of the compound using a chloride free buffer system substituting gluconate for chloride in the respiratory medium. Although the effects of mitoquinone and rotenone appeared to be mildly altered in this non-physiologic buffer, DPI again appeared to block the effect of mitoquinone at all doses of rotenone (figure 5).

### Effect of mitoQ on respiration in intact BAE cells

**Figure 6:**
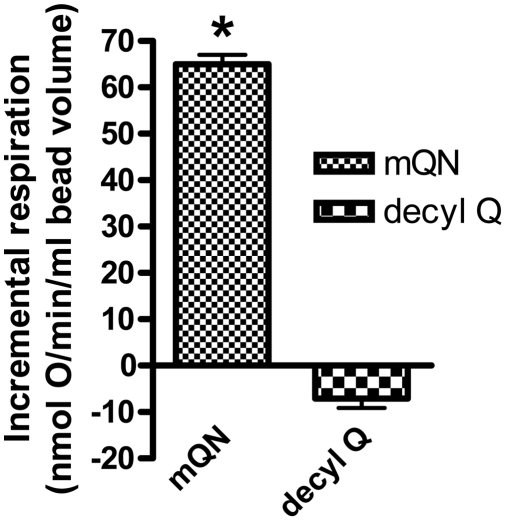
Oxygen consumption by cells perfused on glass beads exposed to mitoquinone (1 µM) or the control compound decylQ (1 µM). Values (mean±SE) represent incremental changes in oxygen consumption (compared to perfusate alone) after addition of mitoquinone or decylQ to the perfusion medium. * p<0.001 compared to control (n = 3).

Given the effects of mitoquinone on respiration in isolated mitochondria we extended these studies to whole cell respiration. This was done by perfusing cells grown on glass beads and measuring the difference in oxygen tension as recorded by electrodes placed proximal and distal to the perfused cells in a manner in which the two electrodes could be precisely calibrated to each other (see supplementary material, Methods S1). The effect of additions to the perfusion medium could then be determined by the change from steady state oxygen consumption prior to and after exposure to the test condition. Addition of mitoquinone resulted in an incremental oxygen consumption rate (beyond that of buffer perfused cells) of 65±2 (mean±SE) nmol O/min/ml bead volume whereas there was essentially no incremental change when the control compound, decylQ was added (figure 6). In each individual experiment comparing mitoquinone to control, the same bead volume and bead preparation were used for both compounds. The mean basal oxygen consumption before addition of these compounds was 56±6 nmol/min/ml bead volume (n = 6) and did not differ between compounds. In additional experiments, which employed separate bead preparations, oxygen consumption after addition of 2.0 µM FCCP was 229±45 nmol/min/ml bead volume (n = 5). A fourth determination of incremental oxygen consumption in the presence of mitoquinone, but with no control, revealed a value of 46 nmol/min/ml bead volume.

### Effect of mitoquinone on fuel selectivity in the intact BAE cell

**Figure 7:**
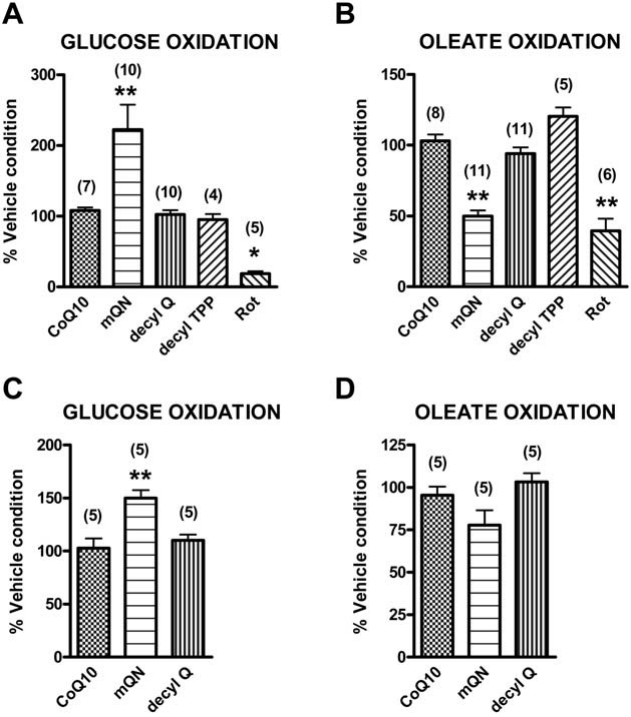
Effect of mitoquinone or control compounds on glucose and oleate oxidation. BAE cells were preincubated for 20 min in the presence of culture medium with 5.5 mM glucose plus 200 µM oleate (panels A and B) or 5.5 mM glucose plus 10 µM oleate (panels C and D). D-[^14^C(U)]glucose (panels A and C) or [1-^14^C]oleic acid (panels B and D) were added at time 0 and cells incubated for 120 min before trapping CO_2_. Final glucose and oleate concentrations after addition of label were 5.6 mM glucose+200 µM oleate (panel A), 5.5 mM glucose+206 µM oleate (panel B), 5.6 mM glucose+10 µM oleate (panel C) and 5.5 mm glucose+15.7 µM oleate (panel D). 1.0 µM mitoquinone or control compounds or 5 µM rotenone (Rot) were added at time −20 min and continued during subsequent incubation. Data are expressed relative to incubation in the presence of vehicle alone, a condition included for study of each cell preparation. Numbers in parentheses designate number of preparations. Data represent mean±SE, * p<0.05 or ** <0.01 compared to non-targeted CoQ10 by one-way ANOVA.

Figure 7 demonstrates the effect of mitoquinone compared to vehicle and control compounds on glucose and oleate oxidation by intact BAE cells determined as CO_2_ production from labeled glucose or oleate. For a given experiment, fat and glucose oxidation were determined in cells grown side by side seeded in individual wells at the same time. Two groups of experiments were performed; first comparing glucose and oleate oxidation in cells at 5.5 mM glucose and 200 µM oleate (before added label) and a second group in cells at 5.5 mM glucose and 10 µM oleate (before added label). Each individual data point actually represents the mean of triplicate determinations in cells grown in separate wells. The absolute values for glucose and oleate oxidation in the presence of vehicle were 4.96±0.85 nmol/well and 216±47 pmol/well in panels A and B, respectively, and 6.45±0.63 nmol/well and 11.8±4.3 pmol/well in panels C and D, respectively.

As indicated, mitoquinone enhanced glucose oxidation (figures 7A and 7C) and inhibited oleate oxidation (figures 7B and 7D), although the effect on oleate oxidation was significant only at the higher oleate concentration. At the higher oleate concentrations, mitoquinone increased glucose oxidation by 222±35% of vehicle compared to little change for the control compounds (CoQ10, decylQ, or decylTPP). Mitoquinone decreased oleate oxidation by 50±4% of vehicle, again compared to essentially no change for the controls. The complex I inhibitor, rotenone, reduced oxidation of both oleate and glucose.

**Figure 8:**
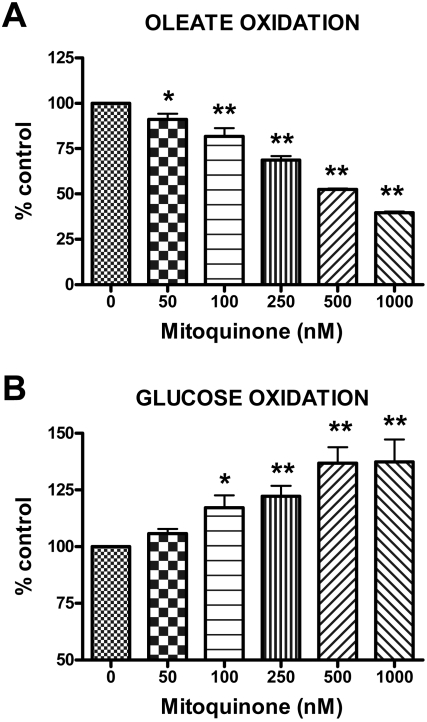
Dose-dependent effect of mitoquinone compared to vehicle on glucose and oleate oxidation. BAE cells were incubated overnight (16 h) in the presence of culture medium with 5.5 mM glucose. [1-^14^C]oleic acid (panel A) or D-[^14^C(U)]glucose (panel B) along with 50 µM oleate were added at time 0 and cells incubated for 120 min before trapping CO_2_. Final glucose and oleate concentrations after addition of label were 5.6 mM glucose+50 µM oleate (panel A) or 5.5 mM glucose+56 µM oleate (panel B). Data are expressed relative to incubation in the presence of vehicle alone. Data represent mean±SE (n = 4 for each determination). * p<0.05 or ** <0.01 compared to vehicle by one-way ANOVA with repeated measures.

We also determined the dose-response characteristics for the effect of mitoquinone on fuel selectivity. These studies were carried out as above except that the oleate concentration was 50 µM and the exposure time to mitoquinone or vehicle was overnight or 18 h. We found a significant dose response relationship extending well down into the nM range (figure 8). The absolute values for glucose and oleate oxidation in the presence of vehicle were 6.21±0.54 nmol/well and 71±18 pmol/well respectively.

### Mitochondrial membrane potential

**Figure 9:**
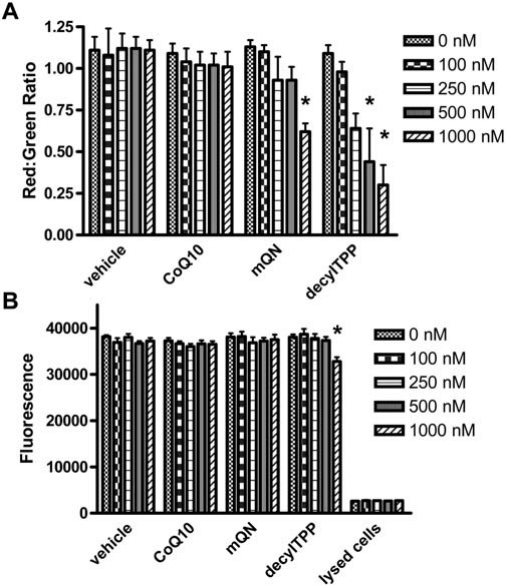
Dose-dependent effects of mitoquinone (mQN), CoQ10, and decylTPP on mitochondrial membrane potential in intact BAE cells and upon cell toxicity. Panel A) Membrane potential estimated as the ratio of red to green fluorescence of the potential sensitive probe, JC-1. Panel B) Cell toxicity estimated as fluorescence generated by reduction of resazurin to resorufin. For both panels, values represent mean±SE, n = 3 for each data point, * p<0.01 compared to the zero concentration by repeated measures ANOVA.

Cells were exposed overnight to 100 nM to 1.0 µM concentrations of mitoquinone, CoQ10, or decylTPP or to vehicle (ethanol) under the same conditions used to study fat and glucose oxidation (figure 9). As shown in figure 9A, mitoquinone and, to a greater extent, decylTPP reduced membrane potential. For mitoquinone, the effect was significant only at the highest dose.

### Cell toxicity

These assays were done in order to test the possibility that mitoquinone or control compounds could induce cytotoxicity, which would raise concern regarding possible non-specific effects on mitochondrial or intact cell fuel utilization or on fuel selectivity. Cells were exposed overnight to 100 nM to 1.0 µM concentrations of mitoquinone, CoQ10, or decylTPP or to vehicle (ethanol), again under the same conditions used to study fat and glucose oxidation (figure 8). We observed no difference in reduction of resazurin to resorufin by BAE cells exposed to any mitoquinone concentration compared to vehicle (figure 9B). Toxicity was observed only at the highest concentration of decylTPP. Conceivably, mitoquinone or control compounds could alter dye reduction. Therefore, we also assessed LDH release from cells treated in this fashion. For all treatments, LDH release was less than 1% of that observed from cells lysed after culture under the same conditions over three separate assays (data not shown).

## Discussion

Our past work [20] showed that mitoQ had prooxidant or antioxidant effects on mitochondria respiring on complex I or complex II substrates, respectively. In this work we expand these studies to add additional information concerning the localization of these effects within the electron transport pathways of complex I. Perhaps, of greater importance, we found that mitoquinone increased mitochondrial respiration selectively on complex I substrates leading to our finding that mitoquinone altered nutrient selectivity at the intact cell level.

Figure 2 confirms our previous findings of the prooxidant effect of mitoquinone during respiration on the complex I substrates glutamate and maleate and provides new information showing that mitoquinone has similar effects on superoxide production in mitochondria fueled by the complex I substrate, pyruvate. This is consistent with the concept that mitoQ to enhances superoxide production at the level of complex I *per se* and is not specific for any particular NADH generating reaction. As shown in figure 2, decylTPP which lacks the quinone moiety does not increase ROS, nor do CoQ or decylQ which lack the cation moiety and do not easily penetrate mitochondria [17], [18].

The major reason for the prooxidant effect of mitoQ is probably redox cycling within complex I. This was suggested by Doughan and Dikalov [21] who recently used inhibitor analysis to propose a model wherein mitoQ undergoes highly efficient redox cycling at two sites within complex I, one proximal and one distal to the putative rotenone binding site, where rotenone is believed to block electron transfer from the N2 iron-sulfur cluster to ubiquinone [21], [36]. Our data (figure 4) are compatible with this two-site model since redox cycling at a single site does not easily account for the inverted U-shaped relationship between ROS production and rotenone in the presence of mitoquinone (figure 4).

As shown in our current (figure 2) and past work [20], both mitoquinone and mitoquinol increase ROS production on complex I substrates consistent with redox cycling properties. Although in all experiments these compounds markedly increase ROS on complex I substrates, we must acknowledge substantial variation in the magnitude of ROS production from experiment to experiment, for example, compare figure 2 to [Fig pone-0004250-g003] figures 4 and 5. We also observed such variation in past studies [20]. We can only speculate regarding the reason for this. We suggest that BAE mitochondria are highly sensitive to these quinone compounds, but that the exact magnitude of ROS generated may be sensitive to subtle differences in conditions from experiment to experiment or in groups of experiments carried out at different times. Perhaps this involves subtle differences in temperature or buffer conditions, in the time it takes to isolate and incubate mitochondria, or the exact nature of mitochondrial preparations from cells grown at different times. In any case, we point out that in all experiments, we compare these quinone/quinol compounds to control conditions within the same mitochondrial preparations, so that the relative effects of these compounds can not be explained by any such variation.

DPI acts proximal to the rotenone site in complex I blocking electron transfer at the FMN site. So the inhibition of the mitoquinone effect at all doses of rotenone by DPI (figure 5) is compatible with mitoquinone action at and/or distal to the FMN site. Existing models of mammalian complex I (which is still an unresolved issue) suggest that FMN (after accepting electrons from NADH), transfers electrons to a chain of iron-sulfur clusters eventually to an N2 site that interacts with ubiquinone [37], [38]. Therefore, if the two site model for mitoquinone cycling is correct (one proximal and one distal to rotenone action at N2) as proposed [39], the DPI data (figure 5) suggest the proximal mitoquinone site is localized at FMN or in the iron-sulfur chain proximal to N2.

In contrast to its prooxidant effect in the presence of complex I substrates, mitoquinone decreased ROS production by mitochondria respiring on succinate (figure 2). Under these conditions considerable electron transport is directed retrograde through complex I, by reverse electron transport, a phenomenon known to be sensitive to membrane potential [20], [22], [40], [41]. In fact, our results show that mitoQ does, in fact, reduce membrane potential (figure 9), so this may explain the effect of mitoQ to reduce ROS generated by mitochondria respiring on succinate. However, as opposed to ROS production generated by reverse electron transport on succinate, reduced membrane potential does not appear to underlie the effect of mitoQ to increase ROS during forward electron transport (respiration on complex I substrates). This is because the compound, decylTPP (mitoquinone minus the quinone/quinol moiety) reduced potential even more than mitoquinone (figure 9) but had no effect to increase ROS during forward electron transport (figure 2).

Our current findings using JC-1 to assess potential as affected by mitoQ in intact cells differs from our past results which utilized the distribution of radiolabeled tetraphenylphosphonium inside and outside isolated mitochondria to assess potential [20]. Using that methodology, we did not see a difference in membrane potential in mitochondria exposed to mitoQ compared to controls. Although we suspected that even a slight reduction in potential might be responsible for the effect of mitoQ to decrease ROS during reverse transport, we concluded that we could not detect it. However, our current data now support this concept.

The substrate-specific effects we observed on ROS production from isolated mitochondria, led us to question whether mitoQ might have similar substrate specific affects on mitochondrial respiration and whether that might translate to intact cell respiration and fuel selectivity at the intact cell level. In fact, mitoquinone did increase mitochondrial oxygen consumption in the presence of complex I but not complex II substrates (figure 3). Of note is that the overall incremental increase in respiration on glutamate plus malate induced by mitoquinone under state 4 conditions was approximately 4 nmoles/mg/min (figure 3). In comparison, the mitoquinone-induced increase in ROS production measured as H_2_O_2_ generated per mg per min was in the picomolar range (figures 2,4,5). Thus, increased radical formation *per se* does not account for the overall increase in mitochondrial respiration suggesting a further effect of mitoquinone. A possible reason for this may be a compensatory increase in respiration as a result of the decrease in membrane potential [42].

As shown in figure 6, mitoquinone increased repiration by intact BAE cells as well as isolated mitochondria. However, of greater interest was the effect of mitoquinone to impart fuel selectivity at the intact cell level, enhancing glucose oxidation while reducing the oxidation of the monounsaturated fatty acid oleate. We had hypothesized that this would be the case based on our observations regarding the substrate specific effect of mitoquinone on mitochondrial ROS production and respiration (see introduction).

The importance of fuel selectivity is underscored by studies indicating that “metabolic inflexibility” or impaired capacity to switch between nutrient utilization, in particular between fatty acid and glucose oxidation, has a pathogenic role in the insulin resistance commonly seen in type 2 diabetes and obesity [25]. Metabolic inflexibility may also be important in cardiac adaptation to stress [23], [24].

According to the classic Randle hypothesis fat and glucose metabolism compete and undergo regulation based on the acetyl-CoA/CoA ratio and citrate concentrations with consequent effects on enzymes regulating glucose and fat metabolism. Later work did not verify this, but placed emphasis on intracellular signaling pathways [43]. More recent metabolomic studies now suggest that enhanced fat metabolism, as seen with high fat feeding, leads to β-oxidation products that exceed mitochondrial capacity restricting the ability to switch to glucose oxidation [25]. That work [25] places emphasis on impaired mitochondrial function suggesting an induced mitochondrial defect but it is unclear what this actually is or exactly how the defect comes into play. Although, our work does not resolve this issue, it does lead to the important conclusion that a primary perturbation at the mitochondrial level (exposure to mitoquinone) can indeed have substantial effects to alter fuel selectivity.

Of course, there are many possible reasons why mitoquinone might alter nutrient selectivity. This will require extensive additional study. Speculative possibilities include mitochondrial redox effects which could affect the state of cytoplasmic reducing equivalents with consequent effects on a myriad of enzyme systems and kinases. These could then alter fuel selectivity at notable steps such as AMPK kinase, pyruvate dehydrogenase, phosphofructokinase, or others. Moreover, it is possible that mitoquinone might have direct effects on mitochondrial proteins such as the pyruvate dehydrogenase complex or the electron transport flavoprotein (ETF)∶ubiquinone oxidoreductase. The later possibility is intriguing since inhibition might impair electron donation by FADH_2_. This might reduce fat oxidation since mitochondrial β-oxidation generates FADH_2_ which is oxidized by theETF∶ubiquinone oxidoreductase donating electrons to the ETS independent of complex I or complex II. Moreover, from the lack of effect of decylTPP+ compared to mitoquinone (figure 7), it appears that the quinone moiety of mitoquinone is essential for its effect on nutrient selectivity.

For several reasons our work suggests that the effects of mitoquinone on ROS production, and respiration occur at complex I. These include the effect of mitoquinone to increase ROS and respiration selectively on complex I substrates (figures 2 and 3), the effect of mitoquinone to decrease superoxide during reverse electron transport to complex I, the interactions of mitoquinone with rotenone and DPI (figure 5), and the knowledge that ROS as detected by DHPA represents matrix superoxide released from complex I (see methods). Our data are not definitive with respect to whether mitoquinone action on complex I is directly related to its effect on intact cell nutrient selectivity. However, given the considerations discussed above regarding the use of mitochondrial reducing equivalents, together with the effects of mitoquinone on mitochondrial respiration, our results at least suggest that complex I effects are involved. Although speculative, our work, also raises the possibility that complex I and endogenous CoQ10 might have similar interactions to affect cell nutrient selectivity. In this regard, there is evidence that the effects of complex I substrates to generate mitochondrial ROS may involve endogenous Q binding sites within complex I [22]. Of course, this issue will require more study and might be aided by better resolution of the structure and function of mammalian complex I in general.

We examined fuel selectivity initially at high and low oleate concentrations, 200 µM and 10 µM, respectively, and subsequently at an intermediate concentration of 50 µM for our dose-response studies. These concentrations were arbitrarily chosen. For perspective, physiologic nonesterified (free) fatty acid concentrations in humans can range widely up to nearly 1 mM depending on nutrient and hormonal status and even higher is some disease states [43]. Of course, oleate represents only a single fatty acids so it is difficult to determine the optimal concentration for study. Oleate in circulating human serum, present among the complex mix of fatty acids, can range roughly 10 to 400 µM [44], [45].

We considered the possibility that the effects of mitoQ on glucose use by intact cells (figures 7 and 8) could reflect a non-specific response to cell toxicity. However, this does not appear to be the case for several reasons. First, we found no evidence for this in our cytotoxic assays. Second, our dose response studies demonstrated effects of mitoquinone extending an order of magnitude or more downward from the 1 µM (or higher) dose used in other reported studies of the cellular actions of this compound [46]–[48] some of which reported a mitoQ induced resistance to apoptosis [46], [47]. Third, although reduced potential might be construed as suggesting cell toxicity, decylTPP (which is also positively charged and differs from mitoQ only in the absence of the Q moiety) did not affect fuel selectivity (figure 7A and 7B) despite greater reduction in membrane potential than mitoquinone (figure 9A). Thus, the effects of mitoquinone are dependent on the quinone moiety and are not explained by membrane potential alone. Finally, we point out that rotenone, which is well known to decrease respiration by complex I inhibition, did not increase glucose oxidation in BAE cells but had the opposite effect (figure 7A and 7B).

Our current work has implications towards the development of mitoQ or related compounds as possible therapeutic agents useful as antioxidants. Possibly this approach might have benefit beyond antioxidant properties and extend to improved ability to use glucose countering the above mentioned effects of diabetes and insulin resistance. It is also important to recognize that although we observed prooxidant rather than antioxidant effects of mitoquinone with complex I substrates, the semiquinone generated by redox cycling of either endogenous CoQ10 or mitoQ may have the beneficial effect of acting as a chain breaking antioxidant [49]. Given the well known vascular nature of the complications of diabetes and the role of atherosclerosis in heart disease and stroke, vascular endothelial cells may be particularly important as targets for antioxidant protection. Moreover, the regulation of endothelial cell glucose use is also an important issue since enhanced glucose consumption is recognized as an adaptive metabolic response to ischemia or hypoxia.

In summary, we provide new data demonstrating that: 1) The prooxidant effects of mitoQ extend to respiration on pyruvate. 2) The prooxidant effects are perturbed by rotenone, but in a complex fashion consistent with action at more than one Q binding site. 3) Importantly, mitoquinone increases mitochondrial respiration in substrate-specific fashion, increases intact BAE cell respiration, and imparts fuel selectivity in the intact cell favoring glucose while inhibiting fat oxidation. 4) MitoQ decreases membrane potential, which likely explains its effect to reduce ROS during reverse electron transport. However, the prooxidant effects of mitoQ and the effects on fuel selectivity are not explained by decreased membrane potential and require the quinone moiety of the compound.

## Supporting Information

Methods S1 Supplemental information
(0.28 MB DOC)
