# Supplementary material for: Mitochondrial Targeted Coenzyme Q, Superoxide, and Fuel Selectivity in Endothelial Cells
Source: PLoS One. 2009 Jan 22;4(1):e4250. doi: 10.1371/journal.pone.0004250 (PMC2621344; doi:10.1371/journal.pone.0004250)
Supplement: Methods S1 — Supplemental information (0.28 MB DOC) [file pone.0004250.s001.doc]

METHODS S1

Assessment of respiration by intact BAE cells perfused on glass beads

Panel A, Respirometer design: Medium, with additives as desired, is directed from the manifold through two identical columns containing glass beads, one with adherent cells and the other (control) with no attached cells. During most operation (valves “a” and “c” closed, valve “b” open), oxygen consumption across the cells is detected by the difference between the upstream and downstream electrode readings. To equally calibrate the two oxygen electrodes, valves “a” and “c” are open and “b” closed so medium is directed (shunted) through the control column wherein no oxygen is consumed. Calibration is done over about two minutes initially, and at intervals during the experimental run. Panel B) BAE cells were perfused with culture medium using the respirometer design above. Upper tracing indicates oxygen content measured at the upstream electrode (before medium is exposed to cells). The lower tracing indicates the downstream oxygen electrode (efflux from cells). O2 is expressed as percent of upstream O2 content calibrated to read 100%. After opening and closing the shunt to calibrate the electrodes to each other at 100% oxygen content, respiration was allowed to plateau before addition of mitoquinone (mQN) and FCCP as indicated. The expected response to uncoupling (FCCP) is an increase in respiration. The downward movement in oxygen content after perfusion with mQN or FCCP indicates an increase in consumption across the perfused cells. The rate of oxygen consumption per bead volume perfused can be calculated as [(% drop in O2 content across the column X partial pressure O2 at atmospheric pressure (nmol/ml) X flow rate (ml/min)] / bead volume (ml). Panel C) Expanded x-axis showing the first 25 minutes of data shown in panel B. Arrows indicate opening and closing of the shunt.

Structures of mitoquinone, CoQ10, and control compounds
